# Refractory Hypothyroidism Due to Improper Storage of Levothyroxine Tablets

**DOI:** 10.3389/fendo.2017.00155

**Published:** 2017-07-10

**Authors:** Salvatore Benvenga, Giampaolo Papi, Alessandro Antonelli

**Affiliations:** ^1^Department of Clinical and Experimental Medicine, Section of Endocrinology, University of Messina, Rome, Italy; ^2^Master Program on Childhood, Adolescent and Women’s Endocrine Health, University of Messina, Rome, Italy; ^3^Interdepartmental Program on Molecular & Clinical Endocrinology, and Women’s Endocrine Health, Azienda Ospedaliera Universitaria Policlinico ‘G. Martino’, Messina, Italy; ^4^Endocrinology Unit of the Northern Area, Azienda USL di Modena, Modena, Italy; ^5^Department of Clinical and Experimental Medicine, Section of Endocrinology, University of Pisa, Pisa, Italy

**Keywords:** levothyroxine, improper storage, replacement therapy, refractory hypothyroidism, environment

## Abstract

**Context:**

A not negligible part of hypothyroid patients on levothyroxine therapy do not normalize serum thyrotropin (TSH) concentrations. “Refractory hypothyroidism,” i.e., a condition characterized by persistently abnormal serum TSH levels despite adequate titration of l-T4 substitution therapy, requires biochemical and instrumental investigation, but no definite etiology is found in up to 15% of cases.

**Objective:**

To report patients presenting with refractory hypothyroidism with proven improper storage of levothyroxine tablets.

**Design:**

Patients on l-T4 substitution therapy referred to three Italian outpatient Clinics of Endocrinology between January 2013 and December 2015 for refractory hypothyroidism were investigated for levothyroxine tablet exposure to humidity, light, and high temperature.

**Results:**

We report eight patients, accounting for approximately 1% of all hypothyroid patients and 5% of those with refractory hypothyroidism in our series. Careful anamnesis disclosed that these patients stored levothyroxine tablets inappropriately. Normalization of serum TSH concentrations was obtained in all cases by simply recommending to store the new levothyroxine tablets away from heat, light, and humidity.

**Conclusion:**

Refractory hypothyroidism linked to improper storage of l-T4 tablets does exist and might be an underrecognized entity. In addition to proper modalities of ingestion of l-T4 tablets, patients need to be instructed on proper modalities of storage, as well.

## Introduction

Hypothyroidism is a common disorder, with a prevalence of approximately 5% and an incidence of approximately 250/100,000 per year in the adult population, but both prevalence and incidence keep rising (1, 2). Consequently, levothyroxine (l-T4) is one of the most prescribed medications. In the United States, l-T4 ranked first in the year 2014 list of top medicines by prescription, because 120 million prescriptions were dispensed (3). This represents a steady 4% annual increment compared with 103, 105, 112, and 117 million in the years 2010, 2011, 2012, and 2013, respectively. In The Netherlands, the prescriptions of l-T4 rose steadily from approximately 305,000 in the year 2005 to 465,000 in 2011 (4). In the United Kingdom, the number of prescriptions has doubled from approximately 7 million in the year 1998 to 19 million in the year 2007 (5). Thus, it is likely that l-T4 prescriptions will increase further worldwide. Even though novel formulations (soft gel capsules, oral solution) have been launched into the marketplace, yet they are not available in all countries (6), and thus the classic formulation of l-T4 for oral use is the tablet.

Replacement therapy of hypothyroidism, which is prescribed by both specialists and general practitioners (7), is monitored by measuring serum thyrotropin (TSH) to ensure it reaches target levels (normalization). Except for the pregnancy setting, the recommended upper limit for serum TSH concentrations is 4.12 mU/L (7), though elderly persons tend to have greater serum levels (2). Based on a recent survey among endocrinologists from all continents, of whom approximately 900 responded, almost 100% of respondents request serum TSH assay to monitor substitutive l-T4 therapy (8). However, 60% of endocrinologists also request serum-free thyroxine, 8% request free triiodothyronine, and 8% request total triiodothyronine. Once the first serum TSH check is performed (mostly between 4 and 8 weeks after initiation of therapy), rechecks are performed every 6 months by half of the respondents, but every 3 months or less by approximately 10% of the respondents (8). Approximately 15–20% of patients taking l-T4 show persistently abnormal serum TSH levels (a condition called “refractory hypothyroidism”), and this problem is frequently addressed by increasing the daily dose of l-T4 (9, 10), with associated frequent requests of the above hormone assays. Ultimately, a thorough diagnostic work-up is necessary to disclose the cause of the problem (9). However, in approximately 15% of such patients, a cause cannot be found (9).

Recent guidelines recommend that “*l**-thyroxine should be stored per product insert at 20–25°C (68–77°F) and protected from light and moisture*” (7). Nevertheless, except for the above review article (9), other reviews (11), guidelines (12), or major textbooks (13) do not mention inappropriate storage among the causes of increased requirement of l-thyroxine. Thus, improper storage remains an overlooked cause. Also, no case reports have appeared to support the above recommendation until the description of grossly improper storage by one of us (14). For this hypothyroid woman, there were multiple factors of improper storage of the T4 tablets. Tablets were removed from the blister, transferred in a transparent vial, and exposed directly to the humidity originating from a humidifier, to the sunlight, and to the high temperature originating from closeness of the tablets to the bedroom heating unit (14). After the patient was instructed to remove all these factors for new l-T4 tablets, serum TSH became entirely normal.

The observation of the above patient (14) underscores the importance of careful history taking as the first step in the management of patients with refractory hypothyroidism (9). Particularly, careful history taking should start with detailed information on modalities of l-T4 tablet storage, the timing of l-T4 ingestion with respect to the timing and type of meals, the liquids used to swallow the tablet, and the use of other drugs/dietary supplements. Upon publication of such case report (14), Salvatore Benvenga was alerted by the coauthors of the present article that they have observed similar cases of elevation of serum TSH, the cause of which could only be improper storage of l-T4 tablets. Indeed, in all these patients, proper storage of new tablets was ensued by normalization of serum TSH.

These patients and the additional patients observed by Salvatore Benvenga are reported here.

## Background

### Patients and Methods

Between January 2013 and December 2015, we have observed eight patients with refractory hypothyroidism in whom, upon careful anamnesis, we suspected improper storage of their l-T4 tablets. Four patients live in Southern Italy (Messina, Sicily), two in Central Italy (Pisa, Tuscany), and two in Northern Italy (Modena, Emilia-Romagna).

Prior to our observation, these eight patients had been managed by their general practitioner, endocrinologist, or both. Based on the experience gained with the patient described previously (14), a careful anamnesis was carried out, starting from a detailed interview on the modalities of storage of the l-T4 tablets. Indeed, in the checklist of the 15 items to take care of in history taking and physical examination of patients with refractory hypothyroidism, the first is to inquire carefully about storage of l-T4 (9). The only abnormality that surfaced at anamnesis in the eight patients was an improper storage of l-T4 tables. Improper storage consisted in taking the l-T4 tablets away from their packages and/or exposing them to heating sources, light and humidity. In all eight patients, we also wished to check the expiration date of the l-T4 tablets that patients were taking, this typically being 18 months from manufacturing. In all cases, the tablets were not expired and were still valid for at least 8 months. Because we were confident that inappropriate storage was the cause of persistent TSH elevation, we immediately suggested proper storage for new packages of l-T4 tablets from the same brand in the same pharmacy, and no changes in the l-T4 daily dose were done to patients. Indeed, we advised the patients to eliminate the l-T4 tablets exposed to heat, light, and/or humidity sources, to take new l-T4 packages from the pharmacy, and to keep new l-T4 tablets within their packages—away from the above cited sources—until the intake time. A thorough diagnostic work-up (9) was planned, should this measure have failed upon the first TSH check 2 months after the beginning of therapy with properly stored new l-T4 tablets.

Three patients observed by Salvatore Benvenga were available for a temperature check of the microenvironment associated with improper storage and the microenvironment associated with proper storage in months representative of the four seasons, using a digital thermometer. External and internal temperatures in the three homes were taken with the same digital thermometer, which was provided by Salvatore Benvenga. No home had air conditioning.

### Statistics

Continuous data are given as mean ± SD and median, while categorical data as percentage. The statistical significance of the difference in serum TSH under the proper storage conditions versus the improper storage conditions was analyzed by the Mann–Whitney test (difference between serum levels) or by the exact Fisher’s text (difference between percentages of target levels). For microenvironment temperatures, the corresponding differences between storages were tested by the one-way analysis of variance (ANOVA).

We considered a *P* value lower than 0.05 as statistically significant, and a *P* value comprised between 0.10 and 0.5 as borderline significant.

### Results

Data are summarized in Table 1.

**Table 1:** Details on changes in thyroid function tests prior to and during l-T4 therapy, with associated daily dosage of l-T4, under conditions of improper l-T4 storage (i.e., before our observation) or proper l-T4 storage (after our observation and recommendations) in the eight patients with refractory hypothyroidism.^a^

	Before and at our observation				After our observation		
Case no., age and sex	L-T4 daily dose [μg/kg bw/day]	FT4 (pg/ml)	TSH (mU/L)	Action taken at our observation	l-T4 daily dose [μg/kg bw/day]	TSH (mU/L)	FT4 (pg/ml)
1, 45 years female	Pretherapy	7.9	**19.6**				
	100 μg/day [1.47]	9.5	**9.5**–**10.8**	l-T4 dose increased to 125 µg [1.84 µg/kg bw/day]			
	125 μg/day [1.84]		**8.6**	l-T4 dose increased to 150 µg [2.20 µg/kg bw/day]	150 μg/day [2.20]	0.51	
	150 μg/day [2.20]		**5.7**	Get a new l-T4 package and store it in a drawer of the wardrobe closet, located in a room with no heating units	100 μg/day [1.47]	2.2	11.6

2, 65 years female	Pretherapy	8.1	**16.2**				
	75 μg/day [1.41]	9.2	**7.3**–**9.4**	Get a new l-T4 package and store in a wardrobe in the bedroom (almost 3 meters away for the heating unit)	75 μg/day [1.41]	3.0–**4.2**	10.9

3, 56 years female	Pretherapy	7.7	**25.8**				
	100 μg/day [1.39]	8.9	**10.9**	l-T4 dose increased to 125 µg [1.74 µg/kg bw/day]			
	125 μg/day [1.74]		**7.6**	150 µg [2.08 µg/kg bw/day]	150 μg/day [2.08]	0.06	
	150 μg/day [2.08]		**4.7**	Get a new l-T4 package and store in a drawer of the nightstand, unexposed to sunlight and located about 1.5 meters from the heating unit	100 μg/day [1.39]	1.8	13.3

4, 71 years female	Pretherapy	N.A.	2.6	l-T4 dose maintained			
	75 μg/day [1.44]		1.0–2.6	Get a new l-T4 package and store it in a drawer of the bedroom commode (located about 3 m from the heating unit).	75 μg/day [1.44]	2.8; 3.0	N.A.
			3.6, **5.9**				

5, 43 years female	Pretherapy	7.3	**27.1**	l-T4 dose increased to 100 µg [1.92 µg/kg bw/day]	100 μg/day [1.92]	2.7	12.4
	75 μg/day [1.44]	10.7	**9.7**–**16.8**	Get a new l-T4 package and locate it into a drawer of another closet in a room with no heating units			
	100 µg [1.92**]**		**9.0**				

6, 52 years female	Pretherapy	6.9	**42.3**	l-T4 dose increased to 175 µg [2.03 µg/kg bw/day]	175 μg/day [2.03]	3.0	13.3
	125 μg/day [1.45]	12.0	**23.7**	Get a new l-T4 package and store it into a drawer of a small room communicating with the kitchen room, but far away from burners and heating unit			
	175 μg/day [2.03]		**7.4**				

7, 78 years male	Pretherapy	7.6	**15**	l-T4 dose increased to 100 µg [1.4 µg/kg bw/day]	100 µg [1.4]	**5.9**	9.4
	75 μg/day [1.0]	7.1	**13**–**21**	Get a new l-T4 package and store it in the drawer of the nightstand			
	100 µg [1.4]		**9.5**				

8, 70 years female	Pretherapy	8.8	**8**	Leave the tablet in the package, and remove it immediately before taking it	125 μg/day [1.90]	2.7–**4.3**	N.A.
		7.3				1.4	
	125 μg/day [1.90]		**11.5**		125 μg/day [1.90]		

			**TSH (mU/L)**		** TSH (mU/L)**		

				Statistics (vs after our observation)^b^			

m ± SD		10.16 ± 4.87		***P* = 3.9 × 10^−7^**	2.87 ± 1.83		

median		9.45			3.0		

>4.12		21/22 measurements (95.4%)		***P* < 0.0001** (OR = 98)	3/17 measurements (17.7%)		

*bw, body weight; FT4, free thyroxine; l-T4, levothyroxine; m, mean; N.A., not available; OR, odds ratio; TSH, thyrotropin*.

*^a^Details on improper and proper modalities of storage. In each of the eight patients, TSH levels above 4.12 mU/L are typed boldface*.

*^b^By Wilcoxon test (m ± SD) or Fisher’s exact test (percentages). FT4 reference values are 8.2–18.4 pg/ml for patient nos. 1–4; 8.0–17.0 pg/ml for patient nos. 5 and 6; 7–15 pg/ml for patient nos. 7 and 8*.

All patients were adult or elderly, and all but one were women. Seven patients were affected by the goitrous variant of Hashimoto’s thyroiditis. The diagnosis of Hashimoto’s thyroiditis was based on elevated antithyroid (ab anti-thyroglobulin and/or anti-thyroperoxidase) autoantibodies and diffusely hypoechoic aspect of the thyroid gland on ultrasound in all cases. One patient (case 4) had previously undergone total thyroidectomy due to a large multinodular goiter with compressive symptoms. The improper storage consisted in exposure of the l-T4 tablets to heating (case nos. 2, 5, 6), humidity (no. 3), both heating and humidity (no. 1), light (no. 7), or both light and heating (no. 4, 8).

In particular, our patients used to store l-T4 tablets as follows:

– Patient 1 in a drug cabinet of water closet, above the heating unit, about 40 cm from both the shower and Jacuzzi; patient 2 in a drawer of the kitchen room, around 20 cm from the burners and owen; patient 3 in a water closet where mold originating from the roof and balcony was evident; patient 4 (previously undergone thyroidectomy for euthyroid benign multinodular goiter) in a transparent glass placed on the nightstand, under both the night light and the abat jour, since 6:00 p.m. until 8:00 a.m.; patient 5 in a drug cabinet of the water closet, above the heating unit; patient 6 in a drawer of the kitchen room, around 40 cm from the burners and owen; patient 7 in a transparent glass placed in the kitchen room, close to the window, since 2:00 p.m. until 6:00 a.m.; patient 8 in a transparent glass placed on the nightstand, close to the heating unit and the light from a night bulb, since 11:00 p.m. until 8:00 a.m.

Improper storage was associated with greater levels of serum TSH and, almost always, with the policy of the family physician and/or endocrinologist to deal with this problem by increasing the daily dose of l-T4. During this time of improper storage, a greater number of TSH assays were requested to monitor therapy. In brief, upon correctly storing the tablets, TSH levels were fourfold lower (*P* < 0.001), and more frequently on target. It should be noted that the proper storage-associated TSH levels of 4.2 mU/L (for the 65-year-old case no. 2), 4.3 mU/L (for the 70-year-old case no. 8), and 5.9 mU/L (for the 78-year-old case no. 7) correspond to the upper normal limit of 4.33 mU/L for the 60–69 year-old reference population, and 5.9 mU/L for the 70–79 year-old reference population (7.50 mU/L for the at least 80-year-old reference population) based on the NHANES III study (2). Finally, if increased prior to our observation, the daily dose of l-T4 could be decreased under proper storage conditions.

As mentioned under Section “Patients and methods,” three patients were available for a temperature check of the microenvironment associated with improper storage and the microenvironment associated with proper storage in months representative of the four seasons (Table 2). Temperatures very close to or above 25°C were measured under conditions of improper storage. Similar temperatures were observed only in summer months under conditions of proper storage. With regard to pertinent weather conditions of the years 2013–2015 for three cities where patients continue to live, the annual relative humidity was 64% (Messina), 72.8% (Pisa), and 78.7% (Modena), with an average of 247 days (Messina) to 352 days of relative humidity ≥ 60% (Modena). Messina was the warmest city, with an average annual temperature of 19.6°C (maximum 22.6°C, minimum 15.9°C), and 94 days of values ≥ 25°C (maximum temperature, 144 days; minimum temperature, 31 days). Like most of Sicily, Messina features an annual average of 300 days and 2700 hours of sunshine compared with an average of 2000 hours in the rest of Italy (15), particularly around 2150 hours in Modena and around 2300 hours in Pisa (16, 17). In sum, Salvatore Benvenga observed twice more cases than either coauthor probably because the weather conditions of Messina mimic the environmental conditions of improper storage better than either Pisa or Modena.

**Table 2:** Temperatures taken at the indicated places in three of the eight patients, all three from Messina, under conditions associated with improper storage (*in italics*) or proper storage.^a^

	Patient no. 1	Patient no. 2	Patient no. 4
External temperature (balcony or window)			

08:00; 16:00; 21:00	17.2; **25.3**; 19.5°C (May)	11.7; 14.5; 12.8°C (March)	15.5; 18.7; 15.8 (April)

08:00; 16:00; 21:00	**25.4**; **32.6**; **29.4**°C (July)	**25.7**; **32.2**; **29.3**°C (August)	24.7; 31.1; 28.5 (August)

08:00; 16:00; 21:00	20.8; **25.2**; 19.5°C (October)	18.1; 22.6; 18.9°C (November)	18.8; 23.4; 19.6 (October)

08:00; 16:00; 21:00	10.7; 15.3; 13.1°C (January)	9.2; 12.7; 10.4°C (February)	11.2; 15.3; 13.0 (January)

Internal temperature, Improper storage	Heating unit Off, *Heating Unit On*	Burners and Owen Off, *Burners and Owen On*	Nightstand lights Off or *On*

Spring (21:00 in pts 1 and 2; 06:00 in pt 4)	24.1°C; N/A (May)	23.5; ***25.3***°C (March)	18.4; ***27.1***°***C*** (April)

Summer (21:00 in pts 1 and 2; 06:00 in pt 4)	**30.7**°C; N/A (July)	**29.6**; ***31.8***°C (August)	**28.0**; ***38.6***°***C*** (August)

Fall (21:00 in pts 1 and 2; 06:00 in pt 4)	20.6°C; N/A (October)	23.1; ***26.7***°C (November)	22.2; ***30.8***°***C*** (October)

Winter (21:00 in pts 1 and 2; 06:00 in pt 4)^c^	15.4°C; ***25.3***°C (January)	22.9; ***25.5***°C (February)	22.4; ***30.3***°***C*** (January)

Mean ± SD [median]	22.7 ± 6.4 [22.3] vs ***25.3*** (n = 1)	24.8 ± 3.2 [23.3] vs ***27.3*** ± *3.0 [26.1]*	22.7 ± 3.9 [22.3] vs ***31.7*** ± *4.9 [****30.5****]*

Statistics (Off vs *On*)	Not done	*P* = 0.29	*P* = 0.029

Internal temperature, Proper storage^b^	Heating unit Off *Heating Unit On*	Burners and Owen Off *Burners and Owen On*	Nightstand lights Off or *On*

Spring (21:00 in pts 1 and 2; 06:00 in pt 4)	22.3°C; N/A (May)	22.0; *22.1*°*C* (March)	17.7; *20.8*°*C* (April)

Summer (21:00 in pts 1 and 2; 06:00 in pt 4)	**29.1**°C; N/A (July)	**29.4**; ***29.4***°C (August)	**27.1**; ***30.2***°***C*** (August)

Fall (21:00 in pts 1 and 2; 06:00 in pt 4)	20.0°C; N/A (October)	19.6; *19.6*°C (November)	19.3; *21.5*°*C* (October)

Winter (21:00 in pts 1 and 2; 06:00 in pt 4)^c^	13.8°C; *22.4*°C (January)	21.7; *21.8*°C (February)	21.6; *22.9*°*C* (January)

Mean ± SD [median]	21.3 ± 6.1 [21.1]; *22.4 (n* = *1)*	23.2 ± 4.3 [21.8]; *23.2* ± *4.3 [21.9]*	21.4 ± 4.1 [20.4]; *23.8* ± *4.3 [22.2]*

Statistics (Off vs *On*)	Not done	*P* = 0.99	*P* = 0.44

Statistics (Off vs Off) Improper vs Proper	*P* = 0.77	*P* = 0.57	*P* = 0.66

Statistics (*On* vs *On*) Improper vs Proper	Not done	*P* = 0.17	*P* = 0.05

*^a^Temperatures ≥ 25.0°C are typed boldface. In patient no. 2, internal temperatures were always taken just before starting to cook or just after ending to cook for dinner. Statistical differences between temperatures tested by one-way analysis of variance (ANOVA)*.

*N/A, not applicable*.

*^b^The closet of patient no. 1 had no heating unit, while the wardrobe in the bedroom of patient no. 2 or the commode in the bedroom of patient no. 4 were located 2.8 or 3.3 m from the heating unit. For patient no. 1, under conditions of proper storage of the l-T4 tablets, heating unit on refers to the nearest heating unit. This was located about 2.5 m away for the wardrobe drawer*.

*^c^Central heating units of the three homes were on, with temperature set at 22.0°C (patient 1 and 4, January) or 21.5°C (patient 2, February). Central heating unit at home of patient no. 2 was on in March, and it was set at 21.5°C*.

## Discussion

Wrong storage of medications at patients’ homes is not rare. Based on an interview of persons living in 49 homes, half of them kept medications in the bedroom or kitchen (18). Moreover, light, temperature and moisture sensitivity is not a thyroid hormone-restricted characteristic (19).

To our knowledge, only one study (reported in abstract form) tested different formulations of l-T4 for their resistance to heat (20). Two novel formulations of l-T4, namely, the soft gel capsule and oral solution, were compared with the classic tablet formulation. The soft gel capsule is a gelatine shield pearl containing l-T4, which is solubilized in glycerol. Oral solution is l-T4 contained in predosed ampoules, with l-T4 solubilized in glycerol and ethanol. The tested l-T4 tablets were of three types: lactose-free but with calcium phosphate; lactose-free, calcium phosphate-free but containing colloidal silicon; lactose-containing. This challenge consisted in keeping each of the five formulations in an incubator at the constant temperature of 25°C for 6 months. At 0, 3, and 6 months, T4 was extracted and measured by variable wavelength detector— high-performance liquid chromatography (HPLC). At the end of the 6 months, the two novel formulations ranked better than tablets.

Approximately 15–20% of patients receiving l-T4 have high serum TSH levels, even when their dose is increased, a condition referred to as “refractory hypothyroidism” that prompts endocrine consultation (9, 10). As detailed in the Section “Introduction,” the issue of improper storage of l-T4 is poorly known. This issue of improper storage is reminiscent of that pertaining to the improper ingestion of l-T4 tablets soon after food intake (most frequently at breakfast) or using coffee as a swallowing liquid or having coffee soon after swallowing the l-T4 tablets with water. The publication of papers reporting patients with refractory hypothyroidism caused by inappropriate intake of l-T4 tablets (21, 22) was of help to both patients and physicians, who have become more aware of such entity (7).

As suspected, the patient reported previously (14) was the classic tip of the iceberg. Together with the eight patients reported here, there are now nine cases described in the literature with refractory hypothyroidism that is likely attributable to inappropriate storage of the l-T4 tablets and consequent degradation of the hormone. In the same 3-year interval of time during which the eight patients came to observation (2013–2015), the authors of the present paper observed a total of approximately 1,500 (or 500 per year) new hypothyroid patients under l-T4 therapy. Twelve percent of these 1,500 patients (180 or 60 patients per year) have refractory hypothyroidism caused by pseudomalabsorption or true malabsorption (e.g., celiac disease, protonic pump-inhibitors, etc.) (9). Hence, in the series reported here, improper storage accounts for approximately 4.5% (8/180) of all cases of refractory hypothyroidism. Also, improper storage would occur at a frequency of approximately 1% (8/1500) of hypothyroid patients taking l-T4 tablet. While we acknowledge that our frequency cannot be universally representative, some projection can be made. For instance, with an estimated 15 million Americans having hypothyroidism, approximately 150,000 of them would have refractory hypothyroidism that could be due to improper storage of l-T4. If their refractory hypothyroidism is managed by progressively increasing the daily dose of l-T4 and if two TSH tests at a cost of $ 50 each are requested to monitor serum TSH, this practice would result in almost $ 8 million excess costs. However, the real rate of refractory hypothyroidism due to improper storage of l-T4 tablets could be higher than 1% of the total hypothyroid patients, because the common strategy of increasing the daily dose of l-T4 could obtain normalization of serum TSH.

The strength of the study is to have collected a series of patients with refractory hypothyroidism that could be attributed to precisely described circumstances in which one or more of three environmental factors were operating: temperature, light, and humidity. Another strength is that the link between inappropriate storage of l-T4 tablets and refractory hypothyroidism was supported by multiple measurements of serum TSH with highly statistically differences compared with the corresponding measurements under conditions of proper storage. Additional support comes from differences in temperatures in the microenvironment associated with inappropriate storage and temperatures associated with appropriate storage. One limitation of our study is not having measured FT4 by high-performance liquid chromatography (HPLC) in both improperly stored and properly stored T4 tablets. A second limitation is not having performed an oral acute loading test with l-T4 for the purpose of excluding pseudomalabsorption. A third limitation is not having tested patients for possible gene polymorphisms that impact on thyroid function tests, though existence of such polymorphisms seems unlikely (see below). Also taking into account the relative high frequency in the population and the possibility of persistent serum TSH elevation upon standard replacement doses of l-T4, one such polymorphism is the Ala92Thr variant of type 2 deiodinase (23).

Thus, it is extremely unlikely that our adult or elderly patients with biochemical (elevated TPOAb and/or TgAb levels) and ultrasound features of thyroiditis had any genetic defect (24), e.g., monoallelic mutations in the TSH receptor (TSHR) gene, causing TSH-resistance and, consequently, refractory hypothyroidism. Indeed, monoallelic inactivating mutations of TSHR are associated with normal or hypoplasic thyroid, which is in contrast with the enlarged thyroid glands detected by US in all our patients. Nonetheless, the thyroidectomized patient was entirely euthyroid prior to thyroidectomy (data not shown). Finally, upon evaluating the past history of patients with Hashimoto’s thyroiditis, we found that four of them had performed thyroid function some years earlier. These tests were performed when one consanguineous relative had developed Hashimotos’ thyroiditis-related hypothyroidism, so they wanted to make sure they were euthyroid and thyroid autoantibody negative. While they were indeed euthyroid, the four patients had elevated levels of TPOAb and/or TgAb (data not shown).

Recently, Van Wilder and co-workers have reported three patients presenting with persistent clinical and biochemical signs of hypothyroidism despite replacement therapy with high doses of levothyroxine (25). Although these three patients denied non-compliance, a peroral 1,000 mcg l-T4 challenge test was positive in all cases, confirming the suspicion of pseudomalabsorption. Daily ingestion of the l-T4 tablets was confirmed by relatives and/or caregivers living with the patients, suggesting the absence of non-compliance as a possibility to explain hypothyroidism in these eight cases.

Another limitation of the present study is that, for humidity, microenvironment indicators were not provided.

## Concluding Remarks

Some hypothyroid patients use to store l-T4 tablets improperly, and this habit causes failure of serum TSH to be normalized. Indeed, when these patients are instructed on the correct storage of l-T4 tablets, serum TSH concentrations normalize. Immediate recognition of l-T4 inappropriate storage avoids mismanagement with increasing daily dose and frequent checks of hormone levels, and also avoids management of patients with unnecessary work-up when patients are referred to specialists. Thus, upon prescription of l-T4 tablets, patients need to be instructed on proper modalities of either ingestion or storage.

## Ethics Statement

This study was carried out with written informed consent from all subjects. All subjects gave written informed consent in accordance with the declaration of Helsinki. Because patients recruited in the study underwent the regular work-up of hypothyroid subjects and were not submitted to life-risk procedures, the study protocol was not submitted to the Ethical Committed of the authors’ Institutions.

## Author Contributions

All the authors contributed equally to recruited patients, and wrote the present work. SB collected data and performed statistical analysis.

## Conflict of Interest Statement

The authors declare that the research was conducted in the absence of any commercial or financial relationships that could be construed as a potential conflict of interest.

The reviewer, DA, and handling editor declared their shared affiliation, and the handling editor states that the process nevertheless met the standards of a fair and objective review.
